# Optimizing Biodegradable Films with Varying Induction Periods to Enhance Rice Growth and Soil Carbon and Nitrogen Dynamics

**DOI:** 10.3390/plants15030358

**Published:** 2026-01-23

**Authors:** Youliang Zhang, Xiaoming Li, Kaican Zhu, Shaoyuan Feng, Chaoying Dou, Xiaoping Chen, Yan Huang, Bai Wang, Yanling Sun, Fengxin Wang, Xiaoyu Geng, Huanhe Wei

**Affiliations:** 1 College of Hydraulic Science and Engineering, Yangzhou University, Yangzhou 225009, China; youliangzhang@yzu.edu.cn (Y.Z.); 18762312192@163.com (K.Z.); syfeng@yzu.edu.cn (S.F.); chydou@163.com (C.D.); xiaoping.chen@yzu.edu.cn (X.C.); 2 Heilongjiang Province Hydraulic Research Institute, Harbin 150080, China; hljskynt@163.com (Y.H.); wangbai1980@163.com (B.W.); sunyanling7980@163.com (Y.S.); 3 State Key Laboratory of Efficient Utilization of Agricultural Water Resources, China Agricultural University, Beijing 100083, China; fxinwang@cau.edu.cn; 4 Agricultural College, Yangzhou University, Yangzhou 225009, China; gengxiaoyuzuishuai@163.com (X.G.); hhwei@yzu.edu.cn (H.W.)

**Keywords:** rice, biodegradable films, rice yield and quality, soil organic carbon, total nitrogen

## Abstract

Polyethylene film (PE) mulching produces substantial “white pollution,” prompting the use of biodegradable film (BF) alternatives, yet their performance in rice systems on Northeast black soils is still uncertain. We compared three BFs with different induction periods (45 d, BF_45_; 60 d, BF_60_; 80 d, BF_80_), PE and a no-film control (CK) to quantify their effects on soil hydrothermal conditions, rice growth, yield, grain quality, irrigation water use efficiency (IWUE) and soil C, N. Results showed that mulching increased soil temperature and soil moisture. Across the growing season, the mean soil temperature at the 0–5 cm depth under PE was 5.5% and 2.2–5.5% higher than that under CK and BFs, respectively. Specifically, compared with CK, PE increased grain yield by 31–77% and IWUE by 75–123%, while BFs improved yield by 25–73% and IWUE by 48–101%. PE only slightly outperformed BF_80_ in yield (by 2.3% in 2023 and 2.1% in 2024) but achieved higher IWUE (11.0–11.7%). Grain chalkiness and sensory scores under BFs were comparable to PE and better than CK. At 0–20 cm, PE increased SOC (2.3–6.8%) and the C/N ratio (0–0.8%) but reduced total nitrogen (TN) (2.7–3.9%) and total carbon (TC) (2.5–3.1%), whereas BFs increased Org-N by 0.4–4.2%, SOC by 2.9–7.1%, and TN by 0.2–0.7%, with BF_80_ showing the greatest stimulatory effect. Overall, BFs—particularly BF_80_—are promising substitutes for PE in black soil rice systems, supporting sustainable rice production with strong application potential.

## 1. Introduction

Global agricultural systems face the dual challenge of meeting rising food demand while reducing environmental impacts under conditions of climate change and resource scarcity [1]. Rice (*Oryza sativa* L.) serves as the primary dietary staple for over half the global population and is among the most water-intensive cereals, requiring 1200–1500 mm of water per growing season—two to three times more water per unit yield than wheat or maize [2]. Despite covering only about 14% of China’s arable land, the black soils (Chernozems) of the Northeast plain contribute nearly one-fifth of the nation’s rice production [3], in addition to 32.8% of maize and 45.8% of soybean output [4]. Owing to their high organic matter content, favorable structure, and strong water-holding capacity, these soils form a major grain production base and a strategic reserve for national food security [5,6,7]. However, their extremely slow formation renders them effectively non-renewable [8,9].

Decades of intensive cultivation, excessive fertilizer inputs, nutrient depletion, and insufficient organic amendments have led to soil acidification, structural degradation, and broader environmental problems that disrupt agroecosystem functioning [10,11]. Soil organic matter in the region has declined by 20–40% over the past three decades, and together with erosion and climate-induced hydrological changes, this deterioration threatens the long-term sustainability of rice production [12]. Empirical evidence indicates that each 0.5% decrease in black soil organic matter can cause more than a 15% yield loss, highlighting the pivotal role of soil fertility in maintaining productivity [13,14]. At the same time, climate change is intensifying hydrological variability, while the continued expansion of irrigated agriculture is exacerbating pressure on limited water resources [15,16], making water scarcity an increasingly binding constraint for rice cultivation in Northeast China. Protecting the black soils of the Northeast plain through effective soil and water conservation practices is therefore crucial for sustaining regional ecosystem health and national food security under mounting climate and water stress [17].

Plastic mulching has become an important practice for improving crop production in water-limited environments [18]. In China, it is used on around 13% of farmland and accounts for nearly 60% of global agricultural plastic film consumption [19]. Mulching conserves soil moisture, moderates soil temperature, suppresses weeds, and reduces competition for water and nutrients, thereby enhancing crop yields and water use efficiency [20,21]. In Northeast China, plastic films are widely applied in rice paddies to alleviate low early-season temperatures, improve seedling establishment, and reduce evaporative losses [22]. However, conventional polyethylene (PE) films degrade very slowly, leaving persistent residues (“white pollution”) that impair soil structure, reduce porosity, increase bulk density, alter microbial communities, and ultimately undermine soil fertility and crop productivity [23,24]. To mitigate these environmental risks, BF has emerged as a promising alternative, capable of decomposing into CO_2_, water, and biomass under field conditions [25]. Recent studies have demonstrated that BFs have the potential to improve soil properties and crop yields [26,27]. Nevertheless, most research has focused on dryland crops such as maize, potato, and wheat in arid or semi-arid regions [28] and systematic evaluations of BFs in cold-region paddy rice systems—particularly those in black soils—remain scarce. Moreover, few studies have combined multiple indicators, including water consumption, crop physiological traits, soil physico-chemical characteristics, and yield and quality, in a single study.

Despite their potential environmental advantages, BF performance in black soil paddy fields is strongly shaped by material properties, degradation dynamics, and local climate. A key knowledge gap concerns how BFs with different degradation induction periods affect the coupled processes of water use, soil hydrothermal conditions, crop physiology, and yield in Northeast China’s rice systems. We hypothesize that biodegradable films with longer induction periods more effectively improve soil thermal–moisture conditions, enhance rice productivity, and promote soil C and N cycling compared to short-period BFs and CK. Accordingly, the objectives of this study were to (1) quantify the effects of different BFs on soil hydrothermal conditions; (2) evaluate how these treatments influence rice growth, yield, quality, and irrigation water use efficiency; and (3) determine the changes in soil organic matter content under various treatments. By integrating agronomic, environmental, and soil quality metrics, this study evaluates the suitability of BFs for paddy rice cultivation in the black soils of Northeast China and provides technical guidance for replacing conventional plastic films in cold-region rice systems in line with national plastic pollution control policies and long-term food security goals.

## 2. Materials and Methods

### 2.1. Experimental Site

The experiment was conducted at the Heilongjiang Water Conservancy Science and Technology Experiment Research Center (45°43′ N, 126°36′ E), located in Xin Nong Town, Daoli District, Harbin City, from May to September in 2023 and 2024. The region is characterized by a temperate monsoon climate, with an average annual temperature ranging from −4 °C to 5 °C. The frost-free period spans 130 to 140 days, and the annual average precipitation is between 400 and 650 mm, with 80% of the rainfall occurring from May to September.

The soil at the experimental site is classified as Haplic Phaeozem (Loamic) in accordance with the IUSS Working Group (2014, 2022), with a depth of 0–100 cm. The soil’s water-holding capacity is 0.36 cm^3^/cm^3^ (by volume), with a bulk density of 1.32 g/cm^3^ and a pH of 7.2. The organic carbon content in the 0–20 cm soil layer is 14.5 g/kg. Furthermore, the available nitrogen (N), available phosphorus (P_2_O_5_), and available potassium (K_2_O) concentrations in the 0–80 cm soil layer are 117.6 mg/kg, 24.1 mg/kg, and 284.7 mg/kg, respectively. Figure S1 presents the precipitation and daily mean temperature.

### 2.2. Experimental Design

The rice variety Daohua Xiang No. 2 was used in the experiment. Planting was carried out on 20 May 2023 and 23 May 2024, with harvesting conducted on 22 September 2023 and 24 September 2024. The effects of black biodegradable film with varying induction periods—45 days (BF_45_), 60 days (BF_60_), and 80 days (BF_80_)—were investigated. In addition, a conventional black plastic film treatment (BN) and an untreated control group (CK) were included, resulting in five treatments, each with three replicates. The experimental design followed a completely randomized design (CRD). The biodegradable film used had a thickness of 0.01 mm and a width of 1.9 m. The procedures for film coverage and rice transplanting are depicted in Figure S2.

Before the rice was planted, the field was soaked for 3 days. Each plot had an area of 27.5 m^2^ (5 m × 5.5 m), consisting of a 5 m-long bed with a width of 1.5 m, separated by a 20 cm wide, 15 cm deep irrigation ditch between each bed. A 30 cm wide, 15 cm deep irrigation ditch was also constructed around the plot perimeter. Embankments, 0.5 m wide and 0.6 m high, with water barriers, were built between plots.

Rice was planted in the north–south direction, with a plant spacing of 12 cm and row spacing of 30 cm, at a density of 277,777 holes per hectare (4 plants per hole). Protective rows were placed around the plot perimeter. Furrow irrigation was used, with water applied to a depth of 3 cm above the bed surface. During the greening stage, a 3 cm water layer was maintained; irrigation during other growth stages was based on tensiometer readings. Irrigation was initiated when the tensiometer (Beijing Shunlong Technology Development Co., Ltd., Beijing, China) reading reached −15 kPa, except during the booting and flowering stages, when the threshold was −10 kPa. After pesticide application (acephate, total active ingredient content 30%; 450–675 g ha^−1^), a 4 cm water layer was maintained for 3 days. Fertilizers applied included urea (N 46%) at 190 kg/hm^2^, slow-release fertilizer (N 44%, with a 60-day release period) at 200 kg/hm^2^, and a mixture of humic acid fertilizer, enzyme bacteria, and Xin Balok fertilizer at 750 kg/hm^2^, all applied as a one-time base application (enzyme-activated organic compound fertilizer (N + P_2_O_5_ + K_2_O ≥ 30%; organic matter ≥ 10%) (Heilongjiang Dafeng Technology Development Co., Ltd., Harbin, China); humic acid bio-organic fertilizer (fulvic acid ≥ 12%; organic matter ≥ 40%; Bacillus megaterium + Paenibacillus mucilaginosus ≥ 0.5 × 100 M/g) (Shandong Quanlin Jiayou Modern Agriculture Co., Ltd., Shandong, China); X-Balok (organic matter content ≥ 30%; nitrogen, phosphorus and calcium content ≥ 5%) (Su Yuan Yuan Bioengineering Co., Ltd., Jiangsu, China), (enzymes 375 kg/hm^2^ + organic fertilizer 300 kg/hm^2^ + Xin Balok 150 kg/hm^2^). The rice growth stages are detailed in Table S1.

### 2.3. Measurement

#### 2.3.1. Meteorological and Soil Temperature Monitoring

Meteorological factors, including temperature and rainfall, were monitored using the automatic weather station at the Heilongjiang Water Conservancy Science and Technology Experiment Research Center. The period from May to September covers the entire rice growth cycle. Total rainfall during this period was 535 mm in 2023 and 414 mm in 2024. Soil temperature was monitored at a depth of 5 cm, in the center of each plot’s bed, avoiding the main root zone of the rice. A button-type soil temperature logger (HOBO MX2201; Onset, Bourne, MA, USA) was installed. The logger’s accuracy is ±0.5 °C, and soil temperature data were recorded hourly. Soil effectively accumulated temperature [29].

(1) $$T_{n}=\sum_{i=1}^{n}\left(X_{i}-10\right)$$

where *T_n_* represents the effective accumulated temperature of the soil (°C), *n* is the total number of days, and *X_i_* is the daily average soil temperature (°C).

#### 2.3.2. Soil Moisture Content Measurement

Soil samples were collected during the rice tillering, jointing and booting, heading and flowering, and maturity stages. A soil auger was used to collect samples vertically between two rows at depths of 0–10 cm, 10–20 cm, 20–30 cm, 30–50 cm, 50–70 cm, and 70–90 cm. Soil moisture content was determined by gravimetric measurements.

#### 2.3.3. Rice Growth Parameters, Yield, and Quality

After the rice regreening stage, plant height was measured every 7 days by selecting 10 fixed hills per plot using a steel tape measure. During the tillering stage, the number of tillers was recorded every 7 days from 10 fixed hills. At maturity (just before rice harvest), 30 hills were randomly selected from each replicate to determine the effective tiller count.

At the tillering, jointing and booting, heading and flowering, and maturity stages, the average number of stems and tillers per plot was used to measure the leaf area from 5 selected hills. Leaf area was determined using the length-width coefficient method. The leaf area index (*LAI*) was calculated as follows [30]:

(2) $$\mathrm{LAI}\;=\frac{\mathrm{Leaf}\;\mathrm{area}\;\mathrm{per}\;\mathrm{hill}}{\mathrm{plant}\;\mathrm{spacing}\;\times \;\mathrm{row}\;\mathrm{spacing}}$$

During the rice tillering, jointing and booting, heading and flowering, and maturity stages, five random hills were selected from each plot. The rice samples were dried using the oven-drying method. The samples were placed in an oven, heated at 105 °C for 0.5 h, after which the temperature was reduced to 80 °C and the samples were dried for 48 h until a constant weight was achieved. The aboveground and belowground dry matter masses were measured using an electronic balance.

At maturity (just before harvest), 30 random hills were selected from each plot to measure the number of effective panicles. From the average number of effective panicles per 30 hills, 5 hills were chosen to measure the number of grains per panicle and the seed-setting rate. After drying the rice (to a moisture content of 11–12%), the yield was determined. A random sample of 1000 grains were selected to measure the 1000-grain weight.

Quality indicators, including milling percentage, brown rice percentage, and chalky rice percentage, were measured according to the national standard GB/T17891-2017 “High-quality Rice” [31]. The amylose and protein content in polished rice was determined using a near-infrared grain analyzer (Inframatic 9500 Plus, Perten Instruments, Hägersten, Sweden). After cooking the polished rice, the rice cooking quality was evaluated using a rice taste meter (STA1A, Satake Corporation, Higashi-Hiroshima, Hiroshima, Japan).

#### 2.3.4. Irrigation Water Use Efficiency [32]

(3) IWUE=Y/I

where *IWUE* represents the irrigation water use efficiency, *Y* is the rice yield (kg/hm^2^), and *I* is the amount of irrigation water applied to the rice crop (mm).

#### 2.3.5. Soil Carbon (C) and Nitrogen (N) Content Measurement

Soil samples were collected from the 0–5 cm and 5–20 cm soil depths in each plot. Three random sampling points were selected from each bed, and the samples from each layer were combined to form two composite samples corresponding to the 0–5 cm and 5–20 cm layers. The samples were air-dried by spreading them on kraft paper bags in a room and then ground using a ball mill. A suitable amount of the ground soil was weighed and analyzed for total carbon (C) and total nitrogen (N) content using a Vario EL Cube elemental analyzer (Elementar Analysensysteme GmbH, Langenselbold, Germany). Approximately 5 g of air-dried soil, passed through a 2 mm sieve, was placed in a 50 mL beaker. Thirty milliliters of 0.2 mol/L hydrochloric acid (HCl) solution were added, and the mixture was thoroughly stirred before standing for 24 h. The sample was then rinsed with deionized water to remove the hydrochloric acid. After drying at 60 °C, the soil samples were ground again using a ball mill. A suitable amount was weighed, and the organic carbon (C) and organic nitrogen (N) content in the soil were determined using the Vario EL Cube elemental analyzer (Elementar Analysensysteme GmbH, Langenselbold, Germany).

### 2.4. Data Analysis

Data were processed using Microsoft Excel 2019, and graphs were generated with Origin 2021. Variance analysis was conducted using SPSS 24 software.

## 3. Results and Analysis

### 3.1. Changes in Soil Temperature and Moisture Content Under Plastic Mulch Coverage

#### 3.1.1. Soil Temperature and Effective Accumulated Temperature Status

**Figure 1:**
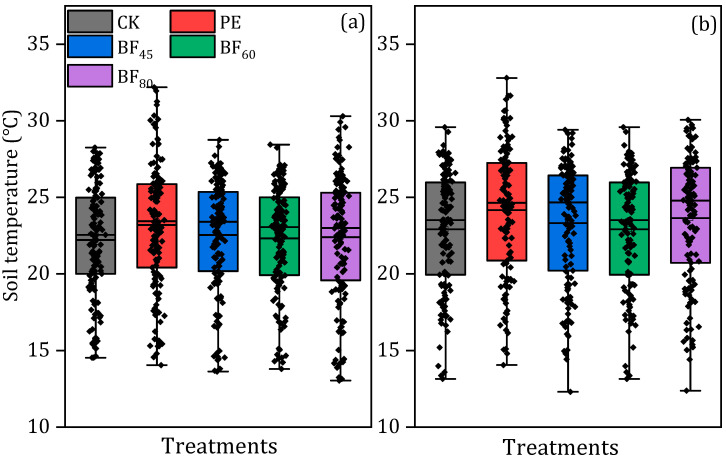
Soil temperature at a 5 cm depth from rice transplanting to the end of the tillering stage under different treatments in 2023 (**a**) and 2024 (**b**). Different letters within the same growth stage indicate significant differences between treatments (*p* < 0.05).

Statistical analysis revealed that the soil water and thermal environment were significantly enhanced by plastic mulch. Among biodegradable films, BF_80_ consistently showed the greatest warming effect, while conventional plastic film (PE) achieved the highest overall soil temperatures (Figure 1). In 2023, the daily mean soil temperature increases under BF_45_, BF_60_, and BF_80_ were 2.1, 1.7, and 2.9 °C, respectively, and 2.1, 2.4, and 2.7 °C in 2024. For daily maximum temperatures, increases were 2.2, 2.5, and 3.5 °C in 2023, and 3.4, 2.8, and 3.4 °C in 2024 for BF_45_, BF_60_, and BF_80_. PE consistently produced the most significant warming, with daily mean temperatures exceeding CK by 3.9 °C in 2023 and 3.4 °C in 2024 (Figure S1). Effective accumulated temperature at 5 cm soil depth during the greening and tillering stages is shown in Table 1. In both years, all BF treatments increased effective accumulated temperature relative to CK, with a more pronounced response during the cooler tillering stage in 2024. In 2023, BF_45_, BF_60_, and BF_80_ increased all-day effective accumulated temperature by 104.1, 77.5, and 103.9 °C, respectively, during greening, and by 102.5, 90.5, and 172.6 °C during tillering. In 2024, the corresponding increases were 75.9, 66.7, and 98.2 °C during greening and 135.3, 173.2, and 152.0 °C during tillering. These patterns indicate that the effects of BFs on soil heat accumulation varied with growth stage and year. During the tillering stage, PE consistently produced higher accumulated temperatures than the biodegradable films, reaching 754.0 °C in 2023 and 736.1 °C in 2024.

#### 3.1.2. Soil Moisture Content

**Figure 2:**
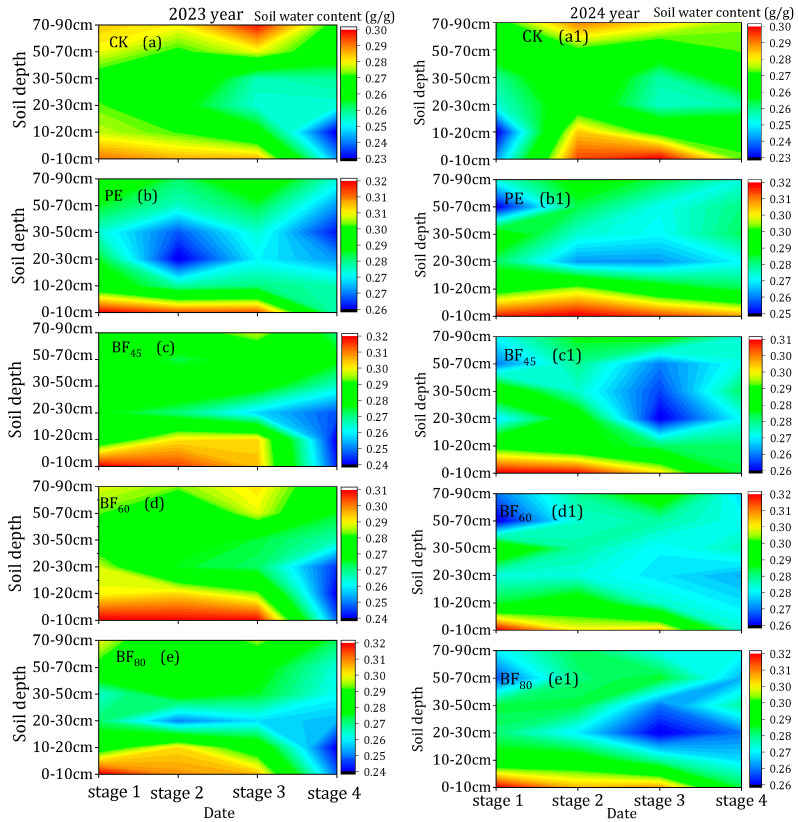
(**a**–**e1**) Soil moisture content at different growth stages of rice over two years. Note: stage 1—tillering stage; stage 2—jointing–booting stage; stage 3—heading–flowering stage; stage 4—ripening stage.

As shown in Figure 2a–e1, plastic mulching markedly altered the distribution of soil moisture across different soil layers (0–90 cm) at different stages in 2023 and 2024. The most significant differences were observed during the tillering stage. In 2023, PE showed a 3.8% increase, while BF_80_ had a 4.1% increase. In 2024, PE increased surface soil moisture by 11.8% relative to CK, compared with 9.9% in 2023. In later stages, the differences between treatments and CK were smaller. For instance, in the ripening stage of 2024, PE increased surface moisture by 4.5% relative to CK. The greatest differences were observed in the early stages, with PE showing 5.6% higher moisture than CK during the jointing–booting stage in 2024 and 5.2% higher in 2023. By the ripening stage, the differences narrowed to 3.5–4.0% in 2023 and 3.9–4.5% in 2024.

### 3.2. Changes in Rice Growth, Yield, Rice Quality, and Irrigation Water Use Efficiency Under Mulching Conditions

#### 3.2.1. Plant Height and Leaf Area

**Figure 3:**
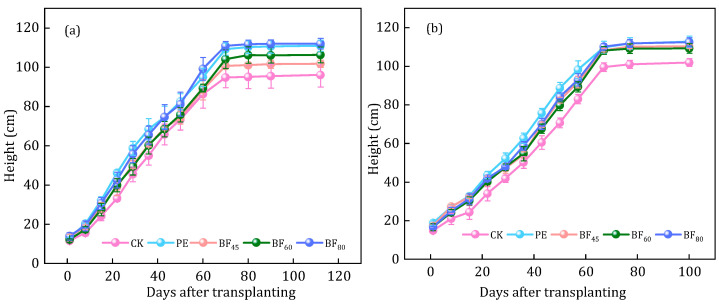
Plant height from transplanting to maturity under different treatments in 2023 year (**a**) and 2024 year (**b**).

Plant height responses to mulching treatments over two years are presented in Figure 3. In both years, plants grown under BFs were taller than CK. In 2023, final plant height reached 101.6 cm (BF_45_), 105.6 cm (BF_60_), and 112.0 cm (BF_80_), while PE attained 110.0 cm, comparable to BF_80_. In 2024, differences among mulched treatments narrowed, with BF_80_, BF_45_, and BF_60_ reaching 111.6, 110.3, and 109.3 cm, respectively. These results indicate that longer induction periods promoted greater plant height, particularly in 2023, although the advantage diminished slightly in 2024. Overall, BF_80_ consistently produced the tallest plants, while PE displayed similar performance by the second year.

Mulching also had a marked effect on canopy development, as reflected in the leaf area index (LAI) shown in Table 2. Across both years, mulching significantly increased LAI at tillering, jointing–booting, and heading–flowering compared with CK. Among biodegradable films, BF_80_ generally maintained the highest LAI across stages, with only minor differences from BF_45_ in 2024. During the heading–flowering stage, LAI under biodegradable films was 27–106% higher than CK. In 2023, LAI values reached 5.06 (BF_45_), 5.55 (BF_60_), and 5.94 (BF_80_), compared with 6.71 under PE; in 2024, the corresponding values were 5.11, 4.96, and 5.18 for BFs, while PE remained highest at 5.90.

#### 3.2.2. Tillering and Dry Matter Accumulation

**Figure 4:**
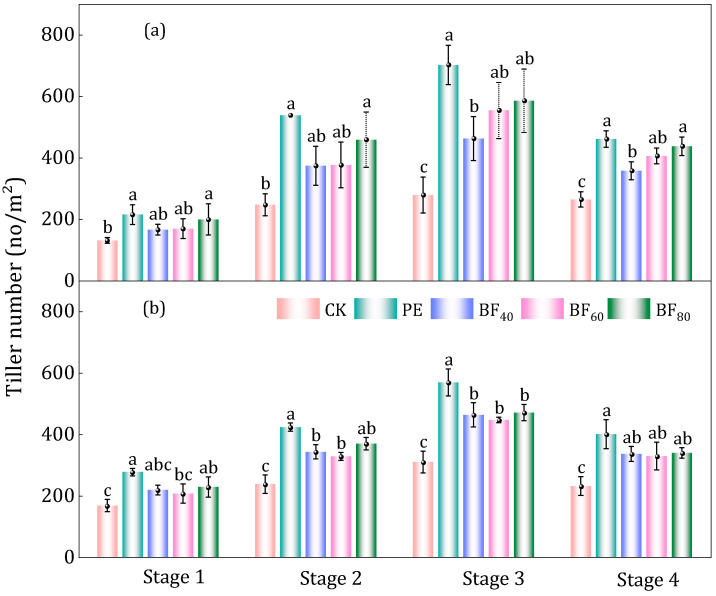
Rice tiller number in 2023 year (**a**) and 2024 year (**b**). Note: Stage 1: Early tillering stage, Stage 2: Mid-tillering stage, Stage 3: Late tillering Stage, Stage 4: Effective tillers at maturity. Note: Letters denote statistical differences among groups. Means sharing the same letter are not significantly different, whereas means with different letters differ significantly.

Mulching treatments significantly increased both the total tiller number during the tillering stage and the number of effective tillers at maturity compared with CK (Figure 4). PE produced the highest tiller numbers and effective tillers. In 2023, BF_45_, BF_60_, and BF_80_ increased tiller numbers by 66%, 99%, and 110% relative to CK (279 tillers/m^2^) and effective tillers by 35%, 54%, and 65% over CK (265 tillers/m^2^), with the magnitude of increase rising progressively with longer induction periods. In 2024, the corresponding increases were 50%, 44%, and 52% for tiller number and 45%, 42%, and 47% for effective tillers relative to CK (309 and 231 tillers/m^2^, respectively); although the differences among BFs were smaller, BF_80_ still produced the greatest enhancement. Across both years, the PE treatment showed the strongest promotion of tillering, with tiller numbers 152% and 84% higher and effective tillers 74% and 73% higher than CK, outperforming all BFs.

**Figure 5:**
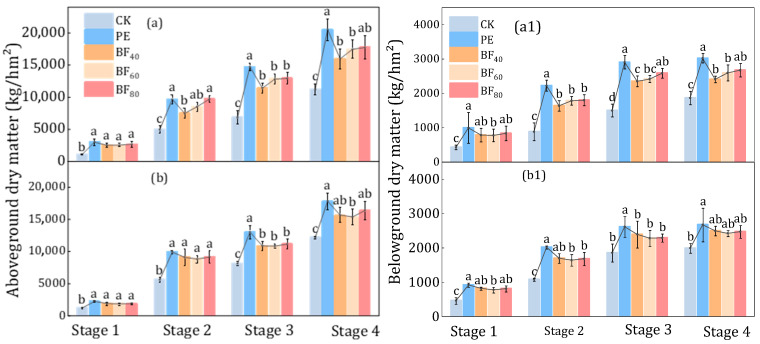
Aboveground and belowground dry matter accumulation of rice in 2023 year (**a**) and 2024 year (**b**). Note: (**a**) 2023 year and (**b**) 2024 year, Stage 1: Tillering stage, Stage 2: Jointing–booting stage, Stage 3: Heading–flowering stage, Stage 4: Ripening stage. Letters denote statistical differences among groups. Means sharing the same letter are not significantly different, whereas means with different letters differ significantly.

Above- and belowground dry matter accumulation is shown in Figure 5a,b,a1,b1. Across both years, mulching significantly increased dry matter compared with CK, with PE consistently producing the highest values. In 2023, BF_45_, BF_60_, and BF_80_ increased aboveground dry matter by 51%, 72%, and 96% at jointing–booting; by 60%, 86%, and 88% at heading–flowering; and by 43%, 56%, and 58% at maturity, respectively, relative to CK. Meanwhile, belowground dry matter increased by 56.7%, 61.1%, and 72.5% at heading–flowering and by 29.1%, 39.2%, and 43.4% at maturity, showing an increasing trend with longer induction periods. In 2024, BF_80_ achieved the highest aboveground dry matter at maturity, 34% greater than CK, whereas belowground dry matter under BF_80_ was slightly lower than under BF_45_. At maturity, aboveground dry matter under PE was 83% and 46% higher than CK in 2023 and 2024, respectively, and its belowground biomass was 7.0–25.5% greater than that of the BFs, confirming the stronger overall biomass accumulation under PE.

#### 3.2.3. Yield, Irrigation Water Use Efficiency (IWUE) and Rice Quality

As shown in Table 3 and Table 4, mulching treatments significantly affected rice yield and irrigation water use efficiency (IWUE) in both 2023 and 2024. Mulching increased yield mainly by increasing the number of productive panicles and grains per panicle, with little change in grain filling percentage and 1000-grain weight. The number of grains per panicle was consistently higher under BFs than under CK, whereas grain filling percentage showed a slight reduction (1.6–2.1% lower than CK in 2023 and 1.2–1.6% lower in 2024), although these differences were not statistically significant (*p* > 0.05). In 2023, yields under BF_45_, BF_60_ and BF_80_ were 54%, 61% and 73% higher than CK, with BF_80_ achieving the maximum increase. In 2024, yield gains of BFs declined to 25–28% above CK, but BF_80_ still maintained the lead among BFs. PE achieved the highest yield overall, with increases of 77% and 31% over CK in 2023 and 2024, respectively. Compared with BF_80_, PE increased grain yield by only 2.3% and 2.1% in 2023 and 2024, respectively, but increased IWUE by 11.0% and 11.7%. Overall, IWUE increased by 48–101% under BFs and by 75–123% under PE relative to CK.

With respect to quality (Table 5), milling performance was similar among treatments. The milled rice rate differed by no more than 0.5%, and the head rice rate under BFs was only 1.0–1.8% higher than CK and close to PE. In contrast, appearance quality improved under mulching. In 2023, BFs reduced chalky grain percentage by 8–51% and chalkiness degree by 19–32% relative to CK. In 2024, the reductions were 9–28% and 3–26%, with BF_60_ giving the lowest values in both years. PE showed milling and appearance quality comparable to BFs. Nutritional and eating quality changed little, protein and amylose contents were almost unchanged, and starch content under BFs increased only slightly (up to 1.5% higher than CK in 2024). Sensory scores were higher under BFs than CK by about 0.3–2.9% in 2023 and 1.5–2.6% in 2024, with BF_60_ and BF_45_ giving the highest scores in each year, but differences among BFs treatments were not statistically significant (*p* > 0.05).

### 3.3. Soil Carbon (C) and Nitrogen (N) Content

Soil C and N contents at 0–5 and 0–20 cm depths are shown in Table 6 and Table 7. In the surface 0–5 cm layer, mulching treatments had little effect on soil organic carbon (SOC), total carbon (TC), or nitrogen fractions, and no significant differences were detected among CK, PE, and the BF treatments. In contrast, at 0–20 cm depth, both PE and BFs slightly increased SOC relative to CK in both years, with SOC under BF treatments being about 3–7% higher than CK and generally comparable to or slightly higher than that under PE, particularly for BF80. Organic N (Org-N) was also marginally higher under BFs than under CK and PE in 2024 (≈4% increase), whereas total N (TN) under BF treatments tended to be slightly lower than CK (about 3–7% reduction), indicating that BFs promoted carbon accumulation more than nitrogen retention. For TC, values under BFs were similar to CK and consistently higher than under PE in 2023, while in 2024 TC decreased across all treatments but remained lowest under PE and slightly higher under BFs. The C:N ratio was close to 12.0 for all treatments in 2023, but in 2024 it increased under BFs by roughly 3–5% compared with CK and PE.

**Figure 6:**
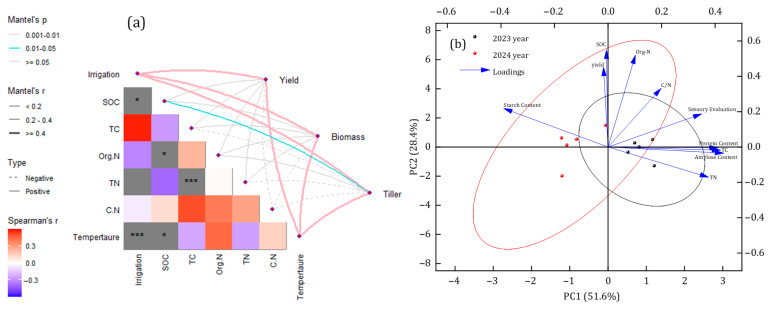
(**a**,**b**) Correlation analysis among soil properties, Yield, Biomass and Tiller; Principal component analysis (PCA) based on Yield, Soil organic carbon (SOC), Total carbon (TC), Organic nitrogen (Org-N), Total nitrogen (TN), Carbon-to-nitrogen ratio (C/N), Protein Content, Starch content, Amylose content, and Sensory evaluation under different mulching conditions. Note: * and *** indicate significant differences at *p* < 0.05 and *p* < 0.001, respectively.

To explore differences among samples from different years and reveal relationships between key variables, we conducted correlation analysis and a principal component analysis (PCA). Temperature and irrigation volume are positively correlated with yield (Figure 6a). Figure 6b displays the PCA results, where PC1 and PC2 explain 51.6% and 28.4% of the total variance in the data, respectively. Soil organic carbon (SOC) and total carbon (TC) showed positive correlations with rice growth and quality traits, indicating that abundant soil organic matter plays a beneficial role in rice development. Meanwhile, organic nitrogen (Org-N) exhibited a negative correlation with rice growth. It should be emphasized that this does not imply that high nitrogen levels per se inhibit rice development but rather that excessive Org-N supply, which can disrupt the optimal soil C:N balance, may induce physiological trade-offs in rice plants (e.g., altered nutrient allocation or reduced resource use efficiency) and thus potentially exert an inhibitory effect on growth. The C/N ratio and total nitrogen (TN) content are also closely related to rice yield and quality, which requires further in-depth study to systematically elucidate the complex relationships between soil properties, yield, and quality throughout the entire rice growth process.

## 4. Discussion

### 4.1. Effect of Film Mulching on Soil Temperature and Soil Moisture Content

Plastic mulching reduces soil evaporation and improves the water use efficiency with limited water resources in arid and semi-arid regions while simultaneously increasing soil temperature and improving the thermal environment for crop growth [33]. Our results indicated that the warming effect of mulch film primarily occurs during the early growth stages of rice (Table 1). However, PE exhibited the most pronounced warming effect, while the warming effect of BFs increased with longer induction periods (Figure 1). Similar conclusions were drawn by [34], who reported that plastic mulching increased soil temperature during all growth stages except at the ripening stage, with significant differences from the control, especially in the 0–0.25 m soil layer. Additionally, significant warming effects from plastic mulching were observed during the seedling and jointing stages of wheat, particularly in the 10 cm and 20 cm soil layers [35].

During the tillering stage, the effective accumulated soil temperature at 5 cm under film treatments showed significant differences compared to CK (Table 1). This is likely because the film suppresses soil evaporation, reducing latent heat loss and altering soil–atmosphere energy partitioning, which contributes to higher surface soil temperature. Additionally, plastic mulching reduces soil evaporation, reallocating more surface energy to sensible heat flux, which contributes to the increase in surface temperature during the early growth stages of crops [36]. However, in the later stages of rice growth, plant canopy coverage and the degradation of BFs gradually diminish their warming and moisture retention performance [37], which explains the smaller differences in soil temperature observed toward ripening.

In the surface soil layer (0–20 cm), mulching markedly enhanced soil moisture compared with CK, with significant differences among cover types (Figure 2a–e1). The film forms a semi-enclosed system with the soil surface, limiting direct evaporation, while condensed water on the underside of the film re-infiltrates into the topsoil. PE consistently maintained the highest moisture content throughout the growing season, whereas BFs retained slightly less moisture, and CK had the lowest values. Among the biodegradable treatments, BF_80_ generally exhibited the best moisture-retention performance, reflecting its longer induction period and slower loss of film integrity. In deeper soil layers (>30 cm), no consistent pattern in moisture among treatments was observed across the two years, suggesting that mulching mainly modifies the hydrothermal regime of the plow layer rather than the subsoil. Previous studies have also shown that mulching improves soil moisture by reducing evapotranspiration; however, during the later growth stages, the degradation of BFs enhances soil evaporation, leading to increased moisture loss [38,39]. Conversely, studies indicated that PE and BFs reduce moisture content in deeper soil layers; the primary reason is that they promote increased absorption of soil moisture by the root system [40]. Moreover, the effectiveness of mulching is also influenced by the type of field, with paddy fields (flooded fields) generally retaining more moisture due to continuous water supply, while dryland fields experience more rapid moisture loss due to lack of irrigation, thus altering the benefits of mulching in different field types. Therefore, a more detailed analysis of the effects of mulching on soil moisture and temperature is necessary to fully understand its potential and limitations across different cropping systems and environmental conditions.

### 4.2. Effect of Mulching on Rice Growth, Yield, IWUE, and Rice Quality

Film mulching improves the soil hydrothermal environment, thereby promoting crop growth and yield. In this study, all mulched treatments increased plant height and canopy development relative to CK, and the effect was most evident in the early growth stages (Figure 3, Table 2). Among the BFs, BF_80_ generally produced the greatest increases in plant height and LAI, whereas PE consistently showed the largest overall enhancement across both years. This indicates that BFs, particularly those with longer induction periods, can approach but do not fully match the growth-promoting effect of PE in the cool conditions of Northeast China. Similarly, Reference [40] found that mulching provides the foundation for main seedling establishment and canopy development. In addition, mulching significantly increased dry matter (Figure 2), with PE consistently outperforming BFs. In 2023, BF_60_ had the greatest increase, while in 2024, BF_80_ was the most effective, showing 34% higher dry matter than CK. PE achieved the highest dry matter, 83% and 46% greater than CK in 2023 and 2024, respectively. Reference [41] indicated that compared to uncovered plots, plastic film coverage significantly increased root biomass and root dry weight by 35.06% and 37.32%, respectively. This is attributed to the mulching creating favorable soil conditions that promote root proliferation, thereby increasing rice yields [42]. In conclusion, PE or BFs, especially those with longer induction periods, can significantly enhance early-stage plant growth and canopy expansion. However, their advantages diminish over time, and PE continues to be the most reliable option for optimizing plant growth in the long term.

The average rice yield over the two study years ranked as PE > BF_80_ > BF_60_ > BF_45_ > CK, indicating that BFs significantly improved yield compared with the control but remained inferior to PE (Table 4). It is noteworthy that temperature had a greater positive effect on production-related indicators, such as tiller number, biomass, and yield, than other soil nutrients (S0C, TC, Org.N, TN, C.N) (Figure 6a); the correlation coefficients were all 0.751 (*p* < 0.05). Thiss is because in Northeast China, where early-season temperatures are relatively low, mulching improves the thermal and hydrological conditions of the soil, thereby facilitating rice emergence and promoting subsequent crop growth and yield [43,44].

Across both years, mulching significantly increased rice yield over CK, mainly through greater productive panicle number. In 2023, PE, BF_80_, and BF_60_ raised panicle number by 55–74%, while in 2024, the increase was 40–73%. Reference [45] also found that compared to PE film, the two BFs increased yields by 8.87% and 6.74%, respectively. Reference [40] indicated that plastic mulching increased average maine yield by 26.74% compared to the CK. This may be due to the provision of favorable water and thermal conditions. In contrast, grains per panicle, grain filling, and 1000-grain weight varied little among treatments. The superior performance of PE and BF_80_ was linked to their ability to maintain favorable soil temperature and moisture during early growth, supporting tiller initiation and survival. BF_45_ degraded earlier, reducing panicle formation. These results confirm that yield gains from mulching in Northeast China rice systems are primarily attributable to enhanced tillering and panicle density rather than changes in grain size or filling. Therefore, studies validated that plastic mulching technology enhances crop yields and improves crop quality. Across two seasons, BF increased IWUE 48–101% versus CK, with longer induction periods generally performing better; PE remained highest (Table 4). Film mulching increases crop WUE by approximately 45–106% across different mulching methods and field conditions in China, while acknowledging potential environmental trade-offs (e.g., residual plastic pollution) [46]. Our ranking of PE ≥ BF > CK mirrors dry-seeded rice trials where both PE and biodegradable films improved water productivity relative to no mulching, although PE typically led to IWUE [47].

Previous research has shown that the application of PBAT/PLA lignin-based biodegradable film in melon cultivation can significantly enhance both yield and fruit quality [48]. This study showed that BFs had minimal effects on milling and nutritional quality but significantly improved appearance and eating quality (Table 5). Milling performance was stable across treatments, while chalky grain percentage and chalkiness degree were markedly reduced under BFs, with BF_60_ (2023) and BF_45_ (2024) showing the lowest values. These results are consistent with reports that hydrothermal conditions during grain filling strongly influence chalk formation [49,50,51]. By moderating soil temperature and moisture, BFs likely enhanced assimilate translocation and starch deposition, thereby reducing chalkiness. Importantly, BFs performed comparably to PE, confirming no disadvantage in processing or appearance quality. Nutritional traits such as protein and amylose contents remained stable, while starch content increased slightly but non-significantly under BFs. Sensory evaluation indicated consistent improvements over CK, with the best performance observed in BF_60_ (2023) and BF_45_ (2024). These enhancements may reflect more favorable starch structural properties and pasting behavior under improved hydrothermal conditions [52,53,54].

Overall, BFs maintained milling and nutritional stability while improving appearance and eating quality to levels comparable with PE mulching. This supports their role as a sustainable alternative for rice cultivation. Future studies should focus on the physiological and molecular mechanisms underlying these effects, particularly the regulation of starch biosynthesis and chalkiness-related genes under variable field environments [55,56]

### 4.3. Effect of Mulching on Soil Carbon (C) and Nitrogen (N) Content

Mulching had little effect on soil C and N in the very surface layer; at 0–5 cm, SOC, TC, and N fractions did not differ significantly among CK, PE, and BF treatments. By contrast, clear effects emerged in the 0–20 cm plow layer (Table 7), where SOC was consistently higher under BFs than under CK (BF_80_ > BF_60_ > BF_45_), and generally not lower than under PE. In 2024, Org-N was slightly higher and TN slightly lower under BFs than under CK and PE, resulting in a modestly higher C:N ratio, especially under BF_80_, indicating relatively greater C accumulation under biodegradable mulching. Overall, longer-induction BFs not only sustain crop production but also promote SOC build-up in the plow layer compared with no mulching and, in some cases, with PE. This is consistent with studies reporting that biodegradable or degradable films foster SOC accrual via enhanced microbial C use efficiency and aggregate protection, and can even reduce SOC decomposition compared with PE under warming scenarios [57]. In parallel, film mulching is known to reshape microbial activity and C/N cycling—mechanisms that plausibly underlie our SOC gains under BF. References [58,59] observed similar increases in soil organic carbon storage under film-covered rice cultivation, largely due to the increased surface soil temperature, which accelerates the mineralization of soil organic matter and promotes rice growth and aboveground biomass. This, in turn, results in greater photosynthate transfer to the roots, leading to substantial increases in organic carbon deposition in film-covered systems [60]. Additionally, BF significantly increased SOC storage and reduced carbon loss in arid agroecosystems. This was due to improved soil temperature and moisture conditions, which enhanced microbial activity and more efficiently converted organic carbon, promoting microbial carbon accumulation [61,62].

Overall, our results demonstrated that the established water-saving advantage of film mulching, while showing that BFs deliver substantial IWUE improvements over CK and tangible SOC benefits, narrowing (though not eliminating) the performance gap to PE. In contexts where plastic pollution and end-of-life costs weigh heavily, BF with longer induction periods offers a pragmatic balance between water efficiency and soil carbon stewardship [56]. In summary, plastic mulching enhances rice yield and improves soil conditions; this phenomenon is also found in maize and peanut [63,64]. Rice production, nutritional quality, and soil environment are significantly correlated. In this study, correlation analysis indicates that SOC had a positive impact on yield, with a correlation coefficient of 0.391 (Figure 6a). PCA analysis also indicated that SOC positively influences yield (Figure 6b). SOC plays an essential role in maintaining soil fertility and boosting crop productivity [65]. Yield is negatively correlated with protein content and amylose content, possibly because increases in grain yield are generally attributed to a dilution effect that reduces protein content [66,67].

## 5. Conclusions

This study compared conventional polyethylene (PE) film with biodegradable films (BFs) of different induction periods in a cold-region paddy rice system on black soils. Both PE and BFs improved soil hydrothermal conditions, enhanced rice growth, and significantly increased yield and IWUE relative to the no-mulch control, with PE giving the highest values and BF_80_ achieving yields only slightly lower than PE. BFs had little effect on milling and nutritional quality but improved appearance and eating quality. BF treatments—especially BF_80_—raised SOC and C:N in the 0–20 cm layer, indicating greater soil carbon accumulation. Overall, BFs with longer induction periods provide a practical compromise between agronomic performance and environmental impact, offering substantial gains in yield, IWUE, and soil carbon while reducing long-term plastic pollution.

## Figures and Tables

**Table 1:** Soil effective accumulated temperature in 2023 and 2024.

Rice Phenological Stages	Treatments	Effective Soil Accumulated Temperature (℃)					
		2023			2024		
		Day	Night	Summation	Day	Night	Summation
Regreening stage	CK	175.9 c	100.8 c	276.7 c	119.7 b	90.4 c	210.1 c
	PE	237.4 ab	167.5 a	404.9 a	170.1 a	122.6 ab	292.7 ab
	BF_45_	227.5 ab	153.3 ab	380.8 ab	168.4 a	117.6 ab	286.0 ab
	BF_60_	211.5 b	142.7 b	354.2 b	162.3 a	114.5 b	276.8 b
	BF_80_	223.2 ab	157.4 ab	380.6 ab	173.7 a	134.6 ab	308.3 ab
Tillering stage	CK	638.1 c	474.9 c	1113.0 c	587.5 b	521.4 b	1108.9 d
	PE	754.0 a	605.1 a	1359.2 a	736.1 a	636.1 a	1372.2 ab
	BF_45_	676.7 bc	538.8 ab	1215.5 ab	663.3 a	580.8 ab	1244.2 c
	BF_60_	676.7 bc	526.8 b	1203.5 b	680.7 a	601.4 ab	1282.1 abc
	BF_80_	717.1 ab	568.5 ab	1285.6 ab	673.2 a	587.7 ab	1260.9 bc
Jointing–booting stage	CK	403.7 a	355.3 a	758.9 a	421.9 ab	391.5 ab	813.4 ab
	PE	367.0 ab	346.9 ab	714.0 b	418.2 abc	386.2 ab	804.4 b
	BF_45_	373.8 ab	348.4 ab	722.2 ab	410.4 bc	384.3 ab	794.7 b
	BF_60_	369.0 ab	345.6 ab	714.6 b	413.5 bc	388.3 ab	801.8 b
	BF_80_	359.7 b	338.4 b	698.1 b	401.5 c	383.2 b	784.7 b
Heading–flowering stage	CK	185.2 a	163.5 a	348.7 a	178.3 ab	163.8 ab	342.1 ab
	PE	160.6 b	154.4 bc	315.1 c	173.0 b	160.2 b	333.2 b
	BF_45_	172.8 ab	158.6 ab	331.4 ab	173.2 b	160.9 b	334.1 b
	BF_60_	169.1 ab	156.8 abc	325.9 bc	174.9 ab	161.7 b	336.5 b
	BF_80_	161.8 b	151.6 c	313.4 c	170.2 b	160.0 b	330.1 b
Ripening stage	CK	447.4 a	358.6 ab	806 a	395.4 ab	341.3 ab	736.7 ab
	PE	413.5 ab	371.9 a	785.4 ab	385.1 abc	343 ab	728.1 abc
	BF_45_	395.4 abc	335 ab	730.4 abc	357.7 bc	318.3 b	676.0 bc
	BF_60_	386.7 abc	328.5 ab	715.2 bc	356.4 bc	315.4 b	671.8 bc
	BF_80_	370.4 bc	323.6 b	694.1 c	358.0 bc	322.8 ab	680.8 bc

Note: Statistical analysis was conducted using Duncan’s multiple range test (*p* < 0.05). Different letters within the same growth stage and column indicate significant differences between treatments (*p* < 0.05), and the same applies hereafter, only referring to treatment differences within the same year and the same rice phenological stage (with year-to-year differences not tested).

**Table 2:** Leaf Area Index (LAI) under different treatments over two years.

Years	Treatments	Leaf Area Index (m^2^/m^2^)			
		Tillering Stage	Jointing–Booting Stage	Heading-Flowering Stage	Ripening Stage
2023	CK	1.31 ± 0.25 b	1.99 ± 0.36 c	2.89 ± 0.27 c	1.59 ± 0.67 c
	PE	3.26 ± 0.37 a	5.92 ± 0.4 a	6.71 ± 0.35 a	4.04 ± 0.44 a
	BF_45_	2.80 ± 0.13 a	4.63 ± 0.29 b	5.06 ± 0.32 b	2.85 ± 0.52 b
	BF_60_	2.76 ± 0.18 a	4.87 ± 0.19 b	5.55 ± 0.31 b	3.38 ± 0.38 ab
	BF_80_	3.11 ± 0.16 a	5.13 ± 0.24 b	5.94 ± 0.31 b	3.42 ± 0.50 ab
2024	CK	1.60 ± 0.13 c	3.15 ± 0.59 c	3.92 ± 0.44 c	2.24 ± 0.20 c
	PE	2.89 ± 0.23 a	5.03 ± 0.44 a	5.90 ± 0.40 a	3.53 ± 0.31 a
	BF_45_	2.38 ± 0.18 b	4.73 ± 0.38 ab	5.11 ± 0.40 b	3.01 ± 0.20 b
	BF_60_	2.27 ± 0.23 b	4.65 ± 0.32 ab	4.96 ± 0.08 b	2.89 ± 0.28 b
	BF_80_	2.45 ± 0.32 b	4.89 ± 0.26 ab	5.18 ± 0.20 b	3.07 ± 0.21 b

Note: Letters denote differences. Identical letters indicate no significant difference between groups; different letters indicate a significant difference.

**Table 3:** Yield and yield components under different treatments.

Years	Treatments	Productive Panicles (×10^4^/hm^2^)	Grains Per Panicle	Grain Filling Percentage (%)	1000-Grain Weight (g)	Yield (kg/hm^2^)
2023	CK	264.8 ± 18.1 c	85.7 ± 3.3 b	94.8 ± 1.7 b	26.2 ± 0.4 a	5485.2 ± 284.1 c
	BN	461.6 ± 18.8 a	100.2 ± 5.1 a	92.8 ± 1.2 ab	24.9 ± 1.1 a	9691.4 ± 915.2 a
	BF_45_	357.9 ± 19.0 b	98.8 ± 4.2 a	93.2 ± 1.8 ab	26.0 ± 0.2 a	8449.8 ± 601.8 b
	BF_60_	406.5 ± 45.3 ab	98.1 ± 9.1 a	93.1 ± 1.3 ab	25.7 ± 0.9 a	8827.2 ± 650.4 ab
	BF_80_	438.0 ± 26.8 a	100.5 ± 8.4 a	92.8 ± 1.5 ab	25.2 ± 0.7 a	9475.4 ± 910.5 ab
2024	CK	231.0 ± 30.8 c	89.3 ± 6.7 b	93.2 ± 1.7 a	26.7 ± 1.2 a	6893.3 ± 333.2 c
	BN	400.3 ± 50.7 a	103.4 ± 3.8 a	91.7 ± 1.5 a	25.9 ± 0.8 a	9030.7 ± 750.8 a
	BF_45_	335.6 ± 24.3 ab	98.3 ± 5.7 ab	91.8 ± 1.6 a	26.0 ± 0.5 a	8760.1 ± 398.4 ab
	BF_60_	328.7 ± 36.1 ab	97.3 ± 7 ab	92.0 ± 2.3 a	26.3 ± 1.3 a	8604.3 ± 414.3 ab
	BF_80_	338.9 ± 16.9 ab	100.8 ± 3.1 ab	91.6 ± 1.4 a	26.2 ± 0.9 a	8847.7 ± 377.6 ab

Note: Letters denote differences. Identical letters indicate no significant difference between groups; different letters indicate a significant difference.

**Table 4:** Irrigation water use efficiency under different treatments.

Treatments	2023			2024		
	Yield (kg/hm^2^)	Irrigation Amount (mm)	IWUE (kg/hm^2^/mm)	Yield (kg/hm^2^)	Irrigation Amount (mm)	IWUE (kg/hm^2^/mm)
CK	5485.2 ± 284.1 c	680	8.1 ± 0.4 c	6893.3 ± 333.2 b	665	10.4 ± 0.5 c
PE	9691.4 ± 915.2 a	535	18.1 ± 1.7 a	9030.7 ± 750.8 a	497	18.2 ± 1.5 a
BF_45_	8449.8 ± 601.8 b	594	14.2 ± 1.0 b	8760.1 ± 398.4 a	547	16.0 ± 0.7 b
BF_60_	8827.2 ± 650.4 ab	589	15.0 ± 1.1 b	8604.3 ± 414.3 a	560	15.4 ± 0.7 b
BF_80_	9475.4 ± 910.5 ab	580	16.3 ± 1.6 ab	8847.7 ± 377.6 a	544	16.3 ± 0.7 ab

Note: Letters denote statistical differences among groups. Means sharing the same letter are not significantly different, whereas means with different letters differ significantly.

**Table 5:** Quality of grinding and appearance, nutritional and taste quality under different treatment conditions.

Years	Treatments	Milling Quality		Appearance Quality		Nutritional Quality		Eating Quality	
		Milled Rice Rate (%)	Head Rice Rate (%)	Chalky Grain Percentage (%)	Chalkiness Degree (%)	Protein Content (%)	Starch Content (%)	Amylose Content (%)	Sensory Evaluation Score
2023	CK	77.4 ± 0.4 a	65.4 ± 2.2 a	0.71 ± 0.11 a	1.08 ± 0.42 a	7.3 ± 0.2 a	79.1 ± 0.2 a	18.5 ± 0.3 a	71.8 ± 1.4 a
	PE	77.5 ± 0.2 a	66.0 ± 3.1 a	0.45 ± 0.20 ab	0.68 ± 0.09 a	7.2 ± 0.1 a	79.2 ± 0.5 a	18.4 ± 0.3 a	72.7 ± 1.6 a
	BF_45_	77.5 ± 0.3 a	66.4 ± 2.1 a	0.39 ± 0.03 ab	0.73 ± 0.26 a	7.2 ± 0.1 a	79.3 ± 0.4 a	18.3 ± 0.2 a	71.0 ± 2.4 a
	BF_60_	77.1 ± 0.5 a	66.4 ± 1.0 a	0.35 ± 0.03 b	0.84 ± 0.13 a	7.1 ± 0.1 a	79.5 ± 0.3 a	18.5 ± 0.1 a	73.4 ± 3.3 a
	BF_80_	77.7 ± 0.7 a	67.4 ± 2.1 a	0.65 ± 0.10 ab	0.88 ± 0.17 a	7.2 ± 0.2 a	79.3 ± 0.5 a	18.4 ± 0.6 a	73.2 ± 4.3 a
2024	CK	75.4 ± 0.5 a	65.8 ± 0.5 a	1.12 ± 0.24 a	2.06 ± 0.47 a	6.9 ± 0.1 a	80.0 ± 0.6 b	17.4 ± 0.3 a	68.3 ± 1.8 a
	PE	76.3 ± 0.7 a	65.9 ± 0.5 a	0.61 ± 0.4 b	1.76 ± 0.42 a	7.0 ± 0.1 a	80.2 ± 0.2 ab	17.4 ± 0.6 a	70.7 ± 0.8 a
	BF_45_	76.9 ± 0.2 a	66.3 ± 0.9 a	0.75 ± 0.31 ab	2.04 ± 0.64 a	7.0 ± 0.1 a	80.4 ± 0.2 ab	17.5 ± 0.6 a	71.8 ± 4.6 a
	BF_60_	75.7 ± 1.8 a	65.8 ± 1.5 a	0.69 ± 0.27 ab	1.52 ± 0.35 a	6.9 ± 0.1 a	80.6 ± 0.2 a	17.4 ± 0.2 a	68.7 ± 4.1 a
	BF_80_	76.9 ± 0.6 a	66.2 ± 1.0 a	1.02 ± 0.36 ab	2.0 ± 0.57 a	7.1 ± 0.2 a	80.3 ± 0.4 ab	17.8 ± 0.6 a	71.0 ± 3.8 a

Note: Letters denote statistical differences among groups. Means sharing the same letter are not significantly different, whereas means with different letters differ significantly.

**Table 6:** Soil C and N content at 0–5 cm depth under different treatments over two years.

Years	Treatments	Org-N (g/kg)	SOC (g/kg)	TN (g/kg)	TC (g/kg)	C/N
2023	CK	1.4 ± 0.03 a	15.4 ± 0.20 a	1.6 ± 0.02 a	18.6 ± 0.06 a	11.9 ± 0.16 a
	PE	1.4 ± 0.03 a	15.7 ± 0.26 a	1.5 ± 0.04 a	18.3 ± 0.70 a	12.0 ± 0.14 a
	BF_45_	1.4 ± 0.05 a	16.1 ± 0.56 a	1.5 ± 0.06 a	18.8 ± 0.35 a	12.2 ± 0.29 a
	BF_60_	1.4 ± 0.03 a	16.1 ± 0.53 a	1.6 ± 0.03 a	18.7 ± 0.33 a	12.0 ± 0.07 a
	BF_80_	1.5 ± 0.03 a	16.2 ± 0.26 a	1.6 ± 0.02 a	18.4 ± 0.19 a	11.7 ± 0.15 a
2024	CK	1.4 ± 0.03 a	16.3 ± 0.21 a	1.5 ± 0.02 a	17.3 ± 0.17 a	11.2 ± 0.12 a
	PE	1.4 ± 0.03 a	16.1 ± 0.18 a	1.5 ± 0.02 a	16.7 ± 1.16 a	11.3 ± 0.78 a
	BF_45_	1.4 ± 0.05 a	16.2 ± 0.58 a	1.5 ± 0.05 a	16.8 ± 0.35 a	11.6 ± 0.54 a
	BF_60_	1.4 ± 0.03 a	16.1 ± 0.19 a	1.4 ± 0.03 a	17.0 ± 0.47 a	11.8 ± 0.16 a
	BF_80_	1.4 ± 0.04 a	16.1 ± 0.62 a	1.4 ± 0.04 a	17.0 ± 0.28 a	11.8 ± 0.49 a

Note: Soil organic carbon (SOC), Total carbon (TC), Organic nitrogen (Org-N), Total nitrogen (TN), Carbon-to-nitrogen ratio (C/N). Letters denote statistical differences among groups. Means sharing the same letter are not significantly different, whereas means with different letters differ significantly.

**Table 7:** Soil C and N content at 0–20 cm depth under different treatments over two years.

Years	Treatments	Org-N (g/kg)	SOC (g/kg)	TN (g/kg)	TC (g/kg)	C/N
2023	CK	2.7 ± 0.08 a	29.7 ± 0.26 a	3.1 ± 0.05 ab	36.8 ± 0.31 a	12.0 ± 0.28 a
	PE	2.7 ± 0.15 a	30.4 ± 0.32 a	2.9 ± 0.11 ab	35.6 ± 1.76 abc	12.1 ± 0.16 a
	BF_45_	2.7 ± 0.11 a	30.6 ± 0.89 a	3.0 ± 0.13 ac	36.1 ± 1.05 ab	12.1 ± 0.27 a
	BF_60_	2.7 ± 0.03 a	30.7 ± 0.52 a	3.0 ± 0.03 abc	36.5 ± 0.81 a	12.0 ± 0.20 a
	BF_80_	2.8 ± 0.02 a	31.3 ± 0.74 a	3.1 ± 0.04 a	36.7 ± 0.56 a	11.8 ± 0.16 a
2024	CK	2.6 ± 0.10 a	29.5 ± 1.36 a	3.0 ± 0.02 abc	17.3 ± 0.17 a	11.2 ± 0.12 a
	PE	2.6 ± 0.02 a	31.5 ± 1.89 a	2.9 ± 0.05 bcd	16.7 ± 1.16 a	11.3 ± 0.78 a
	BF_45_	2.6 ± 0.10 a	30.8 ± 1.02 a	2.8 ± 0.11 d	16.8 ± 0.35 a	11.6 ± 0.54 a
	BF_60_	2.7 ± 0.09 a	31.3 ± 0.94 a	2.9 ± 0.12 cd	17.0 ± 0.47 a	11.8 ± 0.16 a
	BF_80_	2.7 ± 1.31 a	31.7 ± 2.22 a	2.9 ± 0.22 cd	17.0 ± 0.28 a	11.8 ± 0.49 a

Note: Letters denote statistical differences among groups. Means sharing the same letter are not significantly different, whereas means with different letters differ significantly.

## Data Availability

The data presented in this study are available on request from the corresponding author. The data are not publicly available at this stage because the dataset is still being curated and is being used in ongoing manuscript preparation and submission.

## Supplementary Materials

The following supporting information can be downloaded at: https://www.mdpi.com/article/10.3390/plants15030358/s1.
