# Supplementary material for: Optimizing Biodegradable Films with Varying Induction Periods to Enhance Rice Growth and Soil Carbon and Nitrogen Dynamics
Source: Plants (Basel). 2026 Jan 23;15(3):358. doi: 10.3390/plants15030358 (PMC12899773; doi:10.3390/plants15030358)
Supplement: Supplementary file 1 [file plants-15-00358-s001.zip › plants-4089228-supplementary.pdf]

Optimizing rice growth and enhancing soil carbon sequestration via biodegradable mulching films with varying induction periods in cold black soil regions

Youliang Zhang[a], Xiaoming Li[a,*], Kaican Zhu[a], Shaoyuan Feng[a], Chaoying Dou[a], Xiaoping Chen[a], Yan Huang[b], Bai Wang[b], Yanling Sun[b], Xiaoyu Geng[c], Huanhe Wei[c]

[a] College of Hydraulic Science and Engineering, Yangzhou University, Yangzhou 225009, China

[b] Heilongjiang Province Hydraulic Research Institute, Harbin 150080, China

[c] Agricultural College, Yangzhou University, Yangzhou 225009, China

* Corresponding address: x13919750497@163.com (X. Li).

Supplementary Figures and Tables

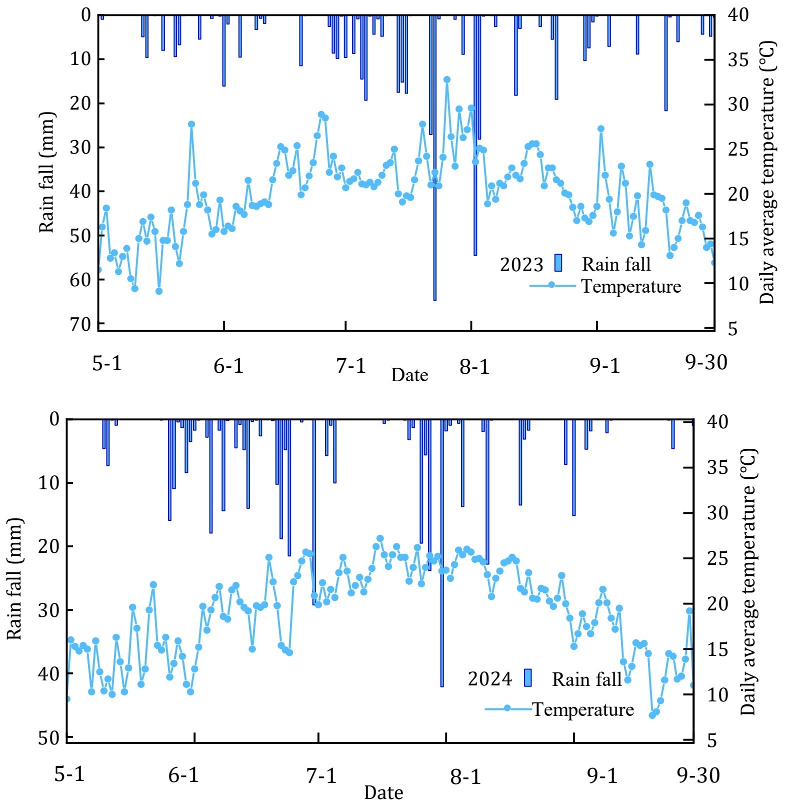

Supplementary Fig.1. Temperature and rainfall during the rice growing season in 2023 and 2024.

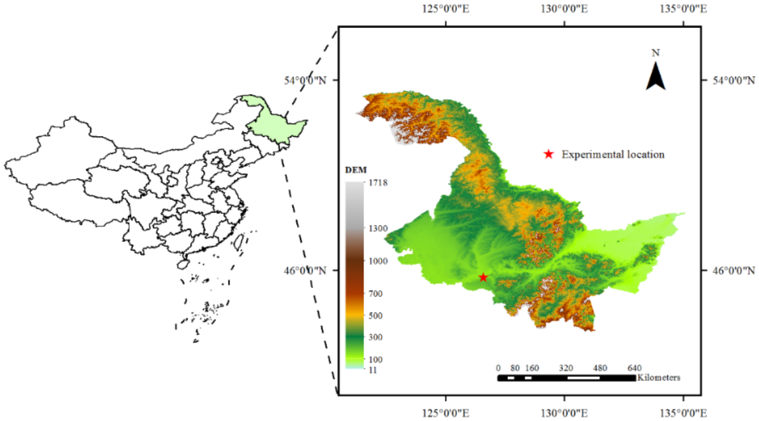

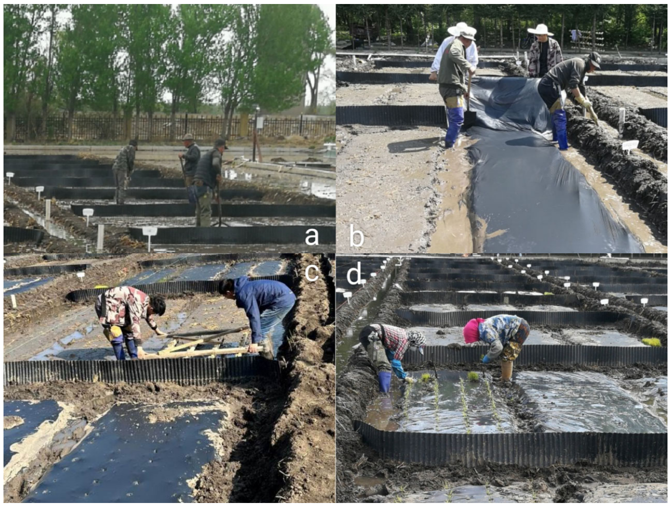

Supplementary Fig.2. Steps of mulching rice operations.

Note: (a) Leveling the bed surface; (b) Mulching; (c) Punching holes; (d) Transplanting rice seedlings.

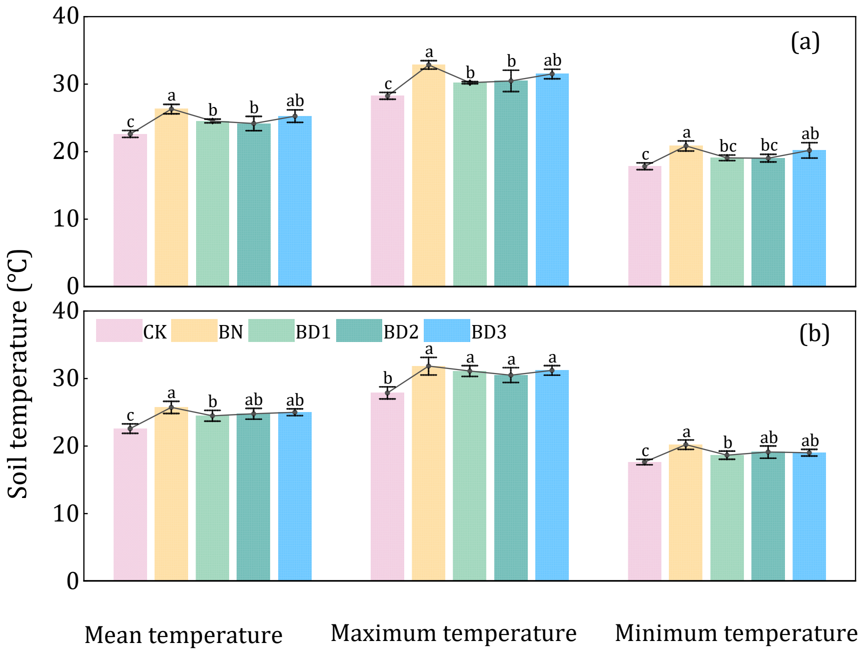

Supplementary Fig.3. Variations in maximum, minimum, and average temperatures in the top 5 cm soil layer of rice under different treatments in 2023 (a) and 2024 (b). Different letters within the same growth stage indicate significant differences between treatments ($p < 0.05$).

Supplementary Table

Supplementary Table 1. Corresponding dates of rice growth stages in 2023 and 2024.

Rice phenological stages	2023	2024
Green-Returning Stage (GR)	May 20th - May 30th	May 23rd - June 4th
Tillering Stage (TL)	May 31st - July 8th	June 5th - July 15th
Jointing-Booting Stage (JB)	July 9th - August 4th	July 16th - August 8th
Heading-Flowering Stage (HF)	August 5th - August 16th	August 9th - August 19th
Ripening Stage (RS)	August 17th - September 22nd	August 20th - September 24th
